# Genetic Implications of HLA-DR and HLA-DQ Genotype on Tobacco Smoking and Oral Submucous Fibrosis

**DOI:** 10.3290/j.ohpd.a44683

**Published:** 2020-07-04

**Authors:** Abhishek Purohit, Bharathi M Purohit, Abin Mani, Ajay Bhambal

**Affiliations:** a PhD Scholar, Department of Dentistry, All India Institute of Medical Sciences, Bhopal, India. Study design, proposal writing, data collection and analysis.; b Assistant Professor, Division of Public Health Dentistry, Centre for Dental Education & Research, All India Institute of Medical Sciences, New Delhi, India.; c Scientist, Centre for Scientific Research and Development, People’s University, Bhopal, India. Lab analysis.; d Dean, Professor and Head, Department of Public Health Dentistry, People’s College of Dental Sciences and Research Centre, Bhopal, India.

**Keywords:** HLA, genetics, tobacco, OSF

## Abstract

**Purpose:**

Integration of genetic information into our understanding of oral diseases has fostered the hope to intervene the disease process among genetically susceptible individuals. Oral submucous fibrosis (OSF) (mainly in the Southeast Asia region) and tobacco smoking are two of the major public health problems the world is facing today. With more and more diseases being associated with alleles of the human leukocyte antigen (HLA), the objective of the study was to explore any genetic association of OSF and smoking behaviour with specific HLA Class II DQB1*0503 and HLA DRB1*0301 alleles.

**Materials and Methods:**

Genomic DNA was extracted from saliva of 64 patients divided into an OSF group, a tobacco smokers group and a control group. This was followed by polymerase chain reaction (PCR) with sequence-specific primer of HLA-DQB1*0503 and HLA DRB1*0301 allele, visualised under 2% agarose gel.

**Results:**

A statistically significant difference was observed between the OSF group and controls in presence of HLA-DQB1*0503 allele, with 84% of the patients showing the presence. Frequency of HLA DRB1*0301 allele was also found to be significantly higher (72%) among OSF patients (p <0.001). Similar results were shown in tobacco smokers with 28% cases showing presence of HLA DRB1*0301 allele and 13 (52%) of them having DQB1*0503 allele (p <0.001).

**Conclusion:**

HLA-DRB1*0301 and HLA-DQB1*0503 are statistically significantly associated with susceptibility to OSF and smoking behaviour.

Tobacco use is a major preventable cause of premature death and is a common risk factor to several general chronic diseases including oral diseases. It is one of the primary risk factors for oral cancer. Approximately one-third of the adult population in the world use tobacco in some form, of whom half will die prematurely. In spite of a wealth of research on epidemiology and genetic implications of tobacco habits, it is still unclear why only few individuals use tobacco and others do not; why some of them quit, while others cannot. Also, some of the important research questions remain unanswered as to why certain nicotine users develop cancer, while others, despite prolonged use of nicotine, do not. The tobacco epidemic is one of the biggest public health threats the world is facing today with 6 million annual deaths reported worldwide and 981,100 deaths occurring annually, with 34.6% or 274.9 million adults in India using tobacco according to the Global Adult Tobacco Survey.^5,23^ Results from the second Global Adult Tobacco Survey 2017 (GATS 2) of India showed that 28.6% of adults aged 15 years and above use tobacco in any form.

Nicotine dependence is a complex behavioural risk factor, widely known to be influenced by environmental factors; but a growing body of evidence attributes interindividual differences in susceptibility to nicotine addiction to various immunologic and genetic factors in different populations.^5,6,8^ Insights into the genetic contributions to tobacco dependence in different populations can potentially help characterise the genetic contribution to both initiation and persistence of smoking^16^ and lead to more effective strategies for management as well as prevention in the future, with an enormous opportunity for public health promotion.^4,17,19^

Oral submucous fibrosis (OSF) is chronic, progressive condition that alters the fibroelasticity of the oral submucosa, predominantly confined to the Southeast Asian region, is now prevalent in the Asian populations of developed countries. Limitation of mouth opening resulting in difficulty in eating is the main presenting feature. It is characterised with a malignant transformation rate of 19% and is therefore a serious threat for global health. The available epidemiological evidence indicates that betel quid chewing or pan masala (a commercial preparation of areca nuts, slaked lime, catechu and undisclosed colouring, flavouring agents, often used with tobacco in smokeless form) is an important risk factor for oral submucosis fibrosis (OSMF) but to date, no conclusive aetiologic agent has been identified.^1,7^

OSF has been associated with the habit of chewing areca quid (AQ) but rarely been described in non-AQ chewing subjects, and severe forms of OSF can be associated with a very short history of chewing. In addition, it has been shown that only 0.1–11% of the AQ chewers develop OSF, with the majority of AQ chewers showing no signs or symptoms.^10,12^ This inconsistency in disease association can be indicative of genetic predisposition and, at present, the specific role that genes play in defining susceptibility remains largely unidentified.^13,22^

These questions have particular relevance in the current scenario when greater attention is focused on harmful effects of tobacco and its products. It has been generally assumed that environmental factors are the primary determinants of use of tobacco products. However, results from adoption, association, trait marker, family and twin studies indicate that genetics factors influence individual differences in vulnerability to tobacco use.^15^ Nearly every disease we know of has a genetic component, although the genetic contribution can vary depending on the disease. The availability and integration of genetic information into our understanding of most common diseases have fostered the hope to unravel genetic causes and provide a mechanism for risk prediction, presymptomatic diagnosis and preventive intervention for individuals susceptible to a variety of complex disorders.^2^ The human leukocyte antigens (HLA), coded by the major histocompatibility complex (MHC), are the cell surface molecules with an important role in controlling the immune system. More and more diseases are being associated with alleles of the HLA region than with any other genetic region.^21^ Hence, interindividual variations in HLA molecules could explain differences in immune responses against microorganisms influencing susceptibility to oral diseases.

These facts prompted us to conduct the study to unmask the possible association of DR and DQ HLA class II alleles among OSF patients and those with tobacco smokers. The objective of the study was to explore the association between HLA Class II DQB1*0503 and HLA DRB1*0301 alleles among OSF patients and smokers.

## Materials and Methods

The study was approved by People’s College of Dental Sciences and Research Centre, Institutional Ethics committee, (DGCI Registration no: ECR/575/Inst/MP/2014 and Project code 2015PHD03) and written informed consent was taken from all the participants prior to the study. The research was conducted in full accordance with the World Medical Association Declaration of Helsinki.

Sample size calculation was based on review of the relevant literature and the formula. According to the estimated prevalence of OSF in India (6%)^14^ and assuming a standard error of 10%, minimum sample size of 21 was calculated. Similarly, with estimated prevalence of cigarette smokers in India being 5.9%^5^ and assuming a standard error of 10%, the minimum sample size of 20 was calculated. Patients visiting the outpatient dental department of two dental colleges under People’s University (ie, People’s College of Dental Sciences and Research Centre and People’s Dental Academy) were invited for participating in the study. On the basis of monthly outpatient’s record and required sample size, study duration of 3 months was decided. At the end of 3 months, 32 OSF patients visited the outpatient department (OPD), of whom 25 subjects agreed to participate in the study, the response rate being 78.12%.

The participants were assigned into three groups: the first group consisted of 25 patients with established OSF without any history of other oral precancerous lesions. In the second group, all the 25 participants were cigarette smokers without the presence of any oral lesion; and the third group had 14 healthy volunteers as controls who were never smokers and without any oral lesion. The general information including age, gender, form of tobacco use, duration and frequency of smoking were recorded.

### Sample Collection

On the day of sample collection, the subjects were asked to vigorously rinse their mouth with a 5 ml sucrose solution (3 %) for 60 s, at least 1 h after tooth brushing. Later, the individuals were asked to rub their tongue on the teeth and oral mucosa and mouthwash of each individual was collected in a 15 ml centrifuge tube. 3 ml of TNE solution (17 mM Tris/HCl (pH 8.0), 7 mM EDTA and 50 mM NaCl) diluted in 66% of ethanol, was added to the centrifuge tube and then subjected to DNA isolation.^1^ The samples collected from the OPD were coded before sending for DNA Analysis to avoid investigator bias.

### DNA Purification and Concentration Measurement

DNA was extracted from epithelial buccal cells collected through salivary sample. The tubes containing the salivary sample were centrifuged followed by washing with 1 ml of TNE (10 mM Tris (pH 8.0), 5 mM EDTA, 0.5% SDS) and overnight incubation. The purity and amount of DNA was determined by spectrophotometry by using the ratio of readings at 260 nm/280 nm. The presence of HLA-DR and DQ alleles was determined by polymerase chain reaction amplification of genomic DNA with sequence-specific primers (PCR-SSP). Later, products of PCR-SSP was electrophoresed on a 2% agarose gel and visualised under ultraviolet (UV) illumination for gel documentation.

### Statistical Analysis

Data was analysed using SPSS (Statistical Package for the Social Sciences) version 21 (SPSS, Chicago, IL, USA). Chi-square test was used to compare categorical variables. Binary logistic regression analysis was applied to estimate odds ratios for the independent variables in predicting the presence of HLA-DR and DQ alleles. Statistical significance was assumed at p value <0.05.

## Results

A total of 64 participants in the age group of 19–25 years were enrolled into the study. The mean age of population was 28 ± 6.83 with the participants being all males except for two females with OSF. The majority of the patients (68%) with OSF used tobacco in smokeless form and the remaining eight patients (32%) used both smokeless and smoked form of tobacco. In the first (OSF) group, the mean duration of using tobacco was 10.52 ± 6.48 years and mean frequency of use was 11.52 ± 5.76 times a day. Similarly, in the second group (tobacco smokers), the mean duration of using a smoked form of tobacco was 8.96 ± 3.84 years and mean frequency of its use was 9.56 ± 2.36 times a day.

Table 1 shows the frequency distribution of HLA-DRB1*0301 and DQB1*0503 alleles among OSF patients, smokers and healthy subjects. Among OSF patients, 18 (72%) of them showed the presence of HLA-DRB1*0301 and 21(84%) of them were positive for HLA-DQB1*0503 allele. Similarly, in the second group, 7 (28%) and 13 (52%) of smokers were positive for the presence of HLA DRB1*0301 and DQB1*0503 alleles, respectively. None of the controls showed the presence of both HLA alleles.

**Table 1:** Frequency distribution of HLA-DQ and DR alleles among the study population

HLA group	Present	Absent	Total
	N (%)	N (%)	
HLA-DQ			
OSF group	21 (84)	4 (16)	25
Tobacco smokers group	13 (52)	12 (48)	25
Control group	0 (0)	14 (100)	14
HLA-DR			
OSF group	18 (72)	7 (28)	25
Tobacco smokers group	7 (28)	18 (72)	25
Control group	0 (0)	14 (100)	14

**Fig 1:**
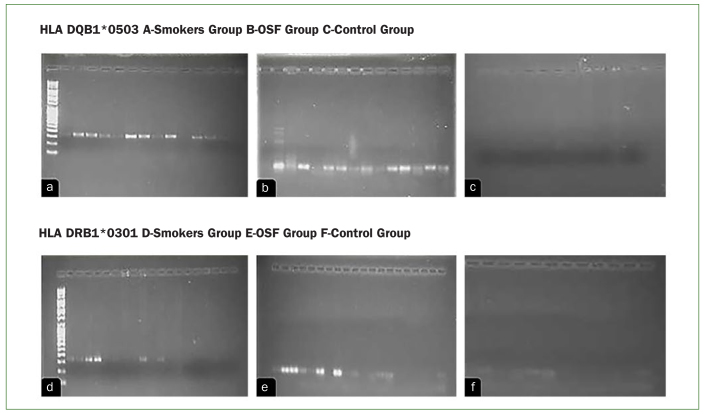
Images (a–f) show the frequencies of HLA-DQ and DR in OSF, tobacco smokers as compared to control group. The brighter band in the picture shows the presence of allele type, while the empty wells indicates the absence.

Figure 1 (a–f) shows the frequencies of HLA-DQ and DR in OSF, smokers as compared to control group. Brighter band in the picture shows the presence of allele type and empty wells indicates the absence.

The association among the study groups with HLA-DRB1*0301 and DQB1*0503 alleles is represented in Table 2. It was seen that statistically significant difference was seen between the OSF patients and the control group (p <0.001) and similarly between the smokers and controls with respect to both the alleles.

**Table 2:** HLA-DQ and DR genomic association among the study groups

HLA groups	Chi-square value	P value
HLA-DQ		
OSF vs controls	25.48	0.00
Tobacco smokers group vs controls	10.92	0.01
OSF vs tobacco smokers group	5.88	0.02
HLA-DR		
OSF vs controls	18.72	0.00
Tobacco smokers group vs controls	4.77	0.04
OSF vs tobacco smokers group	9.68	0.00

Table 3 shows association of HLA-DQ and DR alleles with demographic variables and factors related to use of tobacco among all the study participants. It was seen that age and gender were not significantly associated with both the allelic frequency distribution, whereas use, form and duration of tobacco use were significantly associated with the presence of HLA-DQ and DR allele. The frequency of using tobacco was found to be associated with only HLA-DQ allele.

**Table 3:** Association of HLA-DQ and DR with study variables

Demographic variables	Chi-square value	p value
HLA-DQ		
Age	17.54	0.49
Gender	0.01	0.91
Tobacco consumption	25.48	0.00
Form of tobacco	25.53	0.00
Duration	34.31	0.00
Frequency	30.19	0.00
HLA-DR		
Age	14.19	0.71
Gender	2.46	0.11
Tobacco consumption	18.72	0.00
Form of tobacco	24.36	0.00
Duration	26.79	0.002
Frequency	20.53	0.008

Binary logistic regression analysis was performed with HLA-DQ and DR alleles as dependent variables to establish the contribution of all the tobacco related factors for their presence. Results showed that the form of tobacco use was the only factor significantly associated with presence of HLA-DQ allele (p = 0.03, OR = 3.4). Similarly, with respect to the HLA-DR allele, results showed that the form of tobacco use was the only factor with highest odds ratio of 2.12, although not statistically significant (Table 4).

**Table 4:** Binary logistic regression analysis of study variables on HLA-DQ and DR alleles as dependent variables

Variables	Odds ratio	95% CI	P value
HLA DQ			
Tobacco consumption	0.00	0.00	0.99
Form of tobacco	3.40	1.13–10.28	0.03
Duration	0.99	0.86–1.12	0.83
Frequency	0.96	0.82–1.13	0.65
HLA DR			
Tobacco consumption	0.00	0.00	0.99
Form of tobacco	2.12	0.92–4.9	0.08
Duration	0.92	0.81–1.04	0.17
Frequency	1.06	0.92–1.23	0.39

## Discussion

Genetics has long been linked to play a key role in susceptibility to and protection against a spectrum of oral diseases, with dental researches having an important role in translating genetic technology into oral health promotion. The present study is the first step towards exploring the link between HLA with OSF and smoking behaviour which are fast emerging as global health burdens.

A remarkable number of studies have delved into understanding the role of HLA in determining susceptibility to dental caries and periodontitis, but its genetic role in the etiopathogenesis of OSF has not been well documented to date. Studies by Bagherian et al (2008)^3^ suggested association of HLA-DRB1*04 with susceptibility to ECC and recommended it as a molecular marker for early diagnosis of ECC. Frequencies of HLA-A9 and -B15, HLA-DQA1*03:01, HLA-DQB1*03:02 and HLADQB1*03:05 alleles, as well as that of the HLA-DRB1*04:01 allele, were significantly higher in patients with aggressive periodontitis compared with control subjects among Caucasians and Iranian patients.^12,18^

In the present study we found a statistically significant increase in the allele frequency of HLA-DQB1*0503 and HLA-DRB1*0301 in OSF patients compared to healthy subjects. Hsin-Ming Chen et al (2004) in his study on Taiwanese AQ chewers observed that those with HLA haplotypes B76 are prone to have OSF, although the allele was present only in about 20% of incident cases of OSF.^6^ In our study there was a statistically significant increase in the allele frequency of HLA-DQB1*0503 and HLA-DRB1*0301 in smokers compared with healthy controls without the habit. To date, the main candidate genes that are associated with smoking behaviour are cytochrome P450 enzymes, which implicated in nicotine metabolism. Studies by Linn-Rasker, Verpoort, Michou et al^9,11,20^ have investigated the association between genes and tobacco smoking in rheumatoid arthritis patients and suggested a tendency towards an interaction between the HLA-DRB1*0401 allele and smoking behaviour for anti-CCP positivity. Hence, the present study, to the best of our knowledge, offers pioneering research on HLA alleles, illustrating a very strong association of HLA-DR and DQ with OSF and cigarette smokers.

The results also showed that age and gender were not significantly associated with the allele frequency of both HLA genotypes, which explains that HLA distribution in humans is independent of age and gender. The factors which were significantly associated with HLA were use of tobacco, form and duration and frequency of its use. Results of logistic regression showed that those who used a smokeless form of tobacco were 3.4 times more likely to have the presence of HLA-DQ allele which was statistically significant (p = 0.03) and similarly 2.12 times more likely to have the presence of HLA-DR allele (p = 0.07).

The findings of our study suggested that subjects who carry HLA-DQB1*0503 and HLA-DRB1*0301 genes are significantly associated with tobacco use and presence of OSF. Smoking is a social habit and highly influenced by social factors. However, further research is sought to establish a causal relationship between HLA antigens and tobacco use – and also to conclude that those with the above gene variant have apparently higher likelihood of developing OSF. Sample size could be considered as the limitation of the study; hence, future multicentric longitudinal studies in line with the present observations with larger sample size on populations with varied ethnic background are recommended to establish the causal relationship of HLA alleles with oral diseases.

The available treatment modalities either blunt or cure OSF symptoms but do not touch the underlying cause, nor do they completely control the tobacco epidemic. Hence, the convergence of public health and genetics holds the possibility of improved understanding of the aetiology, prevention, and management of complex oral diseases and could help explain ambiguities in occurrence of oral diseases like OSF and smoking behaviour.^8^ While many challenges lie ahead, the study findings might prove to be a statistically significant step towards understanding the biology behind OSF and smoking behaviour, and provide an insight into utilising these alleles as molecular markers and the development of individual tailored genetic counselling.

### Source of Funding

The study was funded by Colgate-Palmolive (India).
